# Association between Diet Quality and Health Outcomes among Children in Rural Areas of Northwest China

**DOI:** 10.3390/ijerph19137803

**Published:** 2022-06-25

**Authors:** Wanni Yang, Shaoping Li, Yuhe Guo, Yunli Bai, Chengfang Liu

**Affiliations:** 1 China Center for Agricultural Policy, School of Advanced Agricultural Sciences, Peking University, No. 5 Yiheyuan Road, Beijing 100871, China; wanniyang@pku.edu.cn (W.Y.); yuheguo.ccap@pku.edu.cn (Y.G.); 2 School of Economics and Management, Huzhou University, No. 759 Erhuandong Road, Huzhou 313000, China; shaoping66@163.com; 3 Key Laboratory of Ecosystem Network Observation and Modeling, Institute of Geographic Sciences and Natural Resources Research, Chinese Academy of Sciences, No. 11A Datun Road, Beijing 100101, China; baiyl.11b@igsnrr.ac.cn

**Keywords:** diet quality, health outcomes, children, primary caregivers, rural remote areas

## Abstract

This study aims to examine the relationship between diet quality and health outcomes among children in rural remote areas of China. We draw on a cross-sectional dataset of 1216 children from two counties in the Gansu Province in Northwest China. Child health outcomes were assessed with both anthropometric measurements and reports by primary caregivers of the children. Child diet quality was assessed with the diet quality score (DQS) using information from a food frequency questionnaire (FFQ). Our data show the prevalence of stunting and underweight among sample children were 12% and 11%, respectively; 27% of children were reported by their caregivers as unhealthy, and 60% of children had at least one of the seventeen selected non-communicable diseases (NCDs) over the past 14 days. Overall, 780 (72%) children have at least one of the four above-mentioned health problems. Results from logistic regression models show that a higher DQS was significantly associated with a lower likelihood of being stunted and a higher likelihood of being reported healthy after adjusting for confounders. These findings imply that improving child diet quality might be an option when designing interventions to improve child health.

## 1. Introduction

The Sustainable Development Goal (SDG) 3.4 calls for efforts to reduce premature mortality from non-communicable diseases (NCDs) by one-third through prevention and treatment and to promote well-being by 2030 [1]. Although substantial progress has been made in improving child health during the past several decades [2,3], premature mortality from NCDs remains a severe problem in developing countries [4,5]. It is estimated that developing countries experienced 82% of absolute global premature mortality from NCDs in 2020 [6]. In addition, a WHO report shows that a decade of progress in child health could be stalled or reversed by the COVID-19 pandemic which may be the biggest disaster for developing countries in our lifetime [7]. Compared to their urban peers, children living in rural areas suffer more from the pandemic in terms of access to healthy and high-quality food, health care, medicine accessibility, and so on, making the health threats to children more severe than ever before [6,7]. Hence, effective and efficient interventions to reduce NCDs in developing countries are urgently needed, especially among children in their rural areas [8,9].

Previous studies have shown that diet quality is an important factor that may affect NCDs in childhood [10,11]. Diet quality is usually defined as a dietary pattern or an indicator of variety across key food groups linked to the recommended food guidelines [12,13]. A general way of thinking about diet quality is to categorize food items into healthy and unhealthy groups. Examples of healthy food groups include fruits, vegetables, whole grains, fiber, etc. Examples of unhealthy groups include those food items rich in saturated fat, sugar, sodium, etc. Diet quality could be measured by many indices, such as Healthy Eating Index (HEI), Healthy Diet Indicator (HDI), Healthy Food Index (HFI), Recommended Food Score (RFS), Diet Quality Index (DQI), Diet Quality Score (DQS) and Mediterranean Diet Score (MDS) [13]. In general, these diet quality indices could be constructed from a food frequency questionnaire (FFQ), food diary, food guidelines, and so on [14,15,16,17]. In a systematic review by Lazarou and Newby, diet quality was found to be associated with health-related quality of life in children [18]. A healthy diet was found to be significantly associated with micronutrient adequacy, which leads to positive health outcomes [13,15,19], while an unhealthy diet may increase the risks of being stunted and underweight [20,21], which may further hinder health and even socioeconomic success in adulthood [22,23]. Therefore, a better understanding of the relationship between diet quality and child health is of great importance when designing interventions to improve child health. However, to the best of our knowledge, most existing studies mainly focus on children in developed countries or children in cities of developing countries [13]. Few studies have examined the relationship between diet quality and child health in poor rural areas of Northwest China, where children may be more vulnerable to poor diet and malnutrition, which refers to deficiencies or excesses in nutrient intake, imbalance of essential nutrients, or impaired nutrient utilization [6].

The present study aimed to explore the relationship between diet quality and child health by using data from rural areas of the Gansu Province in the northwestern part of China. Gansu is one of the least developed provinces in China in terms of per capita GDP. Children in rural Gansu have been suffering effects of malnutrition such as underweight, stunting, and anemia [24]. Our study focused on children in preschools and primary schools, a key period of child development [9].

## 2. Materials and Methods

### 2.1. Study Population

This study draws on data collected in September 2019 with support from the World Food Program (WFP). The WFP project office selected two then nationally designated poverty counties (D and A) in Gansu Province, Northwest China. The annual per capita disposable income in 2019 for rural residents in D and A was 5906 yuan and 8556 yuan, respectively, which were much lower than that of the national rural average (16,021 yuan, where 1 USD was equivalent to 6.91 Yuan in 2019.). Children in rural Gansu were one of the groups suffering from severe malnutrition problems in China [24]. Within each sampled county, the WFP project office first selected two sample townships, and then selected one preschool and one primary school from each sample township. In total, we have four preschools and four primary schools from four townships across the two project counties. Within each sample preschool or school, all children who attended schools on the survey day were included in the sample, which gave us an original sample of 1216 children. These children come from 76 feed villages of the eight preschools/primary schools. Among these 1216 children, 131 were excluded from the present analysis due to missing information on dietary intake, health outcomes, or other important confounding variables, making the final sample size of 1085 (89%). Our analyses show that there is no systematic difference between those observations with missing information and those without in terms of observable characteristics.

### 2.2. Data Collection

The survey was conducted from 2 September to 30 October 2019 by 65 trained enumerators and four registered nurses in a standardized way. Before the formal field survey, all enumerators and nurses were trained intensively for two days to make sure that they got a standardized understanding of the survey, followed by a practice run in the field. The survey was mainly conducted in Mandarin, except for a few elderly caregivers who could only communicate in the local minority dialect. For these elderly respondents, the questionnaires were administered with the help of school teachers or local officials who volunteered as translators during the field survey.

The survey team collected rich information. For the purpose of the current study, we draw on information from three survey modules. The first module is the diet quality of the sample children, specifically their diet quality score (DQS). The second module examines the health outcomes of sample children, including height and weight measured by the nurses on site, as well as their health status reported by their caregivers. The last module documents the sociodemographic characteristics of children themselves and their mothers, as well as the social-economic status of their households.

### 2.3. Diet Quality

Diet quality was assessed by the unweighted DQS based on responses to a 76-item food frequency questionnaire (FFQ) developed and validated by Liu et al. (2015) [25]. In the survey, each sample child’s primary caregiver was asked to report, on behalf of the child under discussion, the average daily consumption frequency of each of the 76 food items over the last month by choosing from the following four options: never, less than once, once, or more than once. With the above information, we took a three-step approach to construct the unweighted DQS. In the first step, each food item in the FFQ was classified as healthy or unhealthy according to the Chinese Dietary Guideline 2016 and suggestions from the China Nutrition Association [26]. In total, 55 out of the 76 food items were classified as healthy, and the remaining 21 items as unhealthy. In the second step, for each item of “healthy food”, a score of 0 was assigned if a child never ate it on a daily basis over the past month, 1 if less than once, 2 if once, and 3 if more than once. For each item of “unhealthy food”, a score of 0 was assigned if a child never ate it on a daily basis over the past month, −1 if less than once, −2 if once, and −3 if more than once. For scoring purposes, “do not know” was recorded as missing. Finally, the DQS was calculated by adding up the scores that a child got for all the 76 food items. As there are 55 items of healthy food and 21 items of unhealthy food in the FFQ, DQS ranges from −63 to 165, with higher scores indicating better diet quality.

### 2.4. Health Outcomes

Child health was assessed by two sets of measurements. The first set consists of anthropometric ones (height and weight) measured by registered nurses on site. There were 982 children in total with information on anthropometric measurements. Height and weight were then used to construct height-for-age z-scores (HAZ) and BMI-for-age z-scores (BmiAZ), using the WHO Child Growth Standards for children under 5 years old [27] and WHO Growth reference data for children aged 5–19 [28]. Following the guidelines of WHO [27,29], children with HAZ or BmiAZ falling more than two standard deviations below the international means were classified as stunting or underweight, respectively. It is worth noting that the WHO Growth reference data only allow us to screen underweight among children aged less than 120 months. In the case of our study, 249 children were older than 120 months by the time of the survey, which means we were not able to tell whether they were underweight or not in the study.

The other set of health outcomes is composed of the children’s health status reported by their primary caregivers. Specifically, we focus on the health status and whether the child had any symptoms of poor health over the preceding 14 days. In the questionnaire, the primary caregiver was asked to rate the child’s health status by choosing from five options: very healthy, healthy, average, unhealthy, or extremely unhealthy. Based on this information, we created a dummy variable named “Being reported healthy”, which takes the value of one if a child was reported to be “very healthy” or “healthy”, and 0 otherwise (specifically general, unhealthy, or very unhealthy). In addition, primary caregivers were also asked to answer whether their children had any symptoms from a list of 17 nutrition-related NCD symptoms, such as anorexia, diarrhea, decreased appetite, etc. Based on their responses, we created a dummy variable of “Having any NCD symptoms” which takes the value of one if a child happened to have any of the 17 symptoms on the list over the past 14 days and zero otherwise.

### 2.5. Covariates

As the association between DQS and health outcomes might be confounded by socioeconomic factors [15,30,31], we also collected information on child and household socioeconomic and sociodemographic characteristics. At the child level, through interviews with their primary caregiver, we collected data on the child’s gender, ethnicity (Han majority or non-Han ethnic minority), premature status, school level (preschool or primary school), left-behind status, and time spent on TV/mobile. At the household level, we measured the characteristics of the child’s mother (education level and BMI) as well as household socioeconomic status (SES) and the household production diversity score (HPDS). Household SES was proxied by a household asset index based on the household’s possession of durable assets and goods. HPDS was measured by counting the number of nine groups of crops and livestock species that the household produced.

### 2.6. Statistical Analyses

Assuming a design effect up to 0.3 SD, a sample size of approximately 1085 could give a power of 80% at a 5% significance level. Wald tests were used to test differences in child health outcomes by DQS quintiles. Logistic regression models were used to estimate the relationship (with 95% CI) between child health outcomes and their DQS after adjusting for confounding factors (child’s ethnicity, gender, premature status, school level, left-behind status, time spent on TV/mobile, mother’s education level, mother’s BMI status, household SES, HPDS). A two-sided *p*-value < 0.05 was considered statistically significant. Standard errors were clustered at the village level. All analyses were performed using the software STATA^®^ version 15.1 (Stata Corporation, College Station, TX, USA) for Windows.

## 3. Results

### 3.1. Sample Characteristics

Table 1 shows the characteristics of the study sample. Among the 1085 children who participated in the study, 50.41% were girls and the remaining 49.59% were boys. Their mean age was 7.84 (SD 2.82) years. More than half (58.71%) of them were of a minority non-Han ethnicity. About three in ten (32.26%) were preschoolers whereas the remaining 67.74% were pupils. Nearly one in ten (11.74%) children were born premature, and about two-thirds of them (65.07%) were left-behind. In addition, 23.89% of children were reported to spend over one hour on a mobile device or watching TV every day. When it comes to their mothers’ education, about half (49.68%) of them were illiterate, 27.10% of them were literate with less than junior high school education, and 23.22% of them obtained at least a junior high school education. As to their mothers’ BMI status, 64.61% of them were in the normal weight category, 14.01% of them were underweight, and the remaining 21.38% were overweight. Of the five selected durable assets, their households possess 2.2 pieces on average. In terms of HPDS, 88.48% were low with HPDS less than four, and 11.52% were medium with HPDS greater than three but less than seven. None of the sample households have an HPDS greater than six.

Our data show that the mean DQS of sample children was 28.57 (SD 11.29). The prevalence of stunting and underweight were 12.32% and 10.50%, respectively. According to reports by their primary caregivers, 72.81% of children were healthy, and 60.46% of them had at least one of the 17 selected NCD symptoms during the past 14 days. A total of 780 (71.89%) children had at least one of the four above-mentioned health problems, namely stunting, underweight, being reported as unhealthy, or having any NCD symptoms.

### 3.2. Association between DQS and Health Outcomes

Table 2 describes the health outcomes of children by their DQS quintiles. Results from the Wald test show that children with higher DQS tended to be less likely to be stunted, and more likely to be healthy. Specifically, when a child’s DQS increases from the bottom quintile to the top quintile, his/her likelihood of being stunted decreases from 19.03% to 7.33%, and the likelihood of being reported healthy increases from 66.15% to 81.09%. In contrast, results from the Wald test do not show any obvious pattern in being underweight or having any NCD symptoms over the preceding 14 days by DQS quintiles.

Table 3 presents the results from logistic regression models. For each health outcome variable, we first report results from unadjusted models, followed by results from adjusted models where child characteristics (gender, ethnicity, premature status, education level, left-behind status, and time spent on TV/mobile), mother characteristics (education and BMI status), and household characteristics (Household SES, and HPDS) were adjusted. Consistent with results from the descriptive analysis above, results from regression analyses also showed that higher diet quality was associated with a lower likelihood of being stunted (OR = 0.96, 95% CI: 0.95–0.98, *p* < 0.01) and a higher likelihood of being reported as healthy (OR = 1.03, 95% CI: 1.02–1.05, *p* < 0.01) by their primary caregivers. Adjusting for additional confounding variables did not alter our findings above (*p* < 0.05, both in stunting and being reported healthy). However, the association between DQS and underweight/having any NCD symptoms could not be concluded in our study (*p* > 0.05).

As the results of adjusted models show in Table 3, girls were more likely than boys to be underweight (OR = 1.43, 95% CI: 1.02–2.01) and have NCD symptoms over the preceding 14 days (OR = 1.27, 95% CI: 1.03–1.58). Compared with pupils, preschoolers were less likely to be underweight (OR = 0.59, 95% CI: 0.38–0.90). Meanwhile, compared with children who live with both parents around, left-behind children were less likely to be stunted (OR = 0.58, 95% CI: 0.39–0.85), but more likely to have any NCD symptoms over the preceding 14 days (OR = 1.47, 95% CI: 1.04–2.09). When it comes to maternal education, the results revealed that compared to children whose mothers had no formal education, children whose mothers had graduated from junior high school and above were more likely to be reported as healthy (OR = 2.31, 95% CI: 1.32–4.06).

## 4. Discussions

Using a cross-sectional dataset from rural areas of Northwest China, we found the prevalence of stunting among sample children in the study site was 12.32%, which was much higher than that of the national average (0.99%) in 2020 [32]. A recent study conducted in South Central China found a similar prevalence of stunting among preschoolers at 10.64% [33]. We also found that the prevalence of those underweight was 11% in the sample children, which was also higher than the national average (1.19%) in 2020 [32]. These results imply that efforts are needed to reduce the stunting and underweight prevalence among preschoolers and pupils in rural remote areas in Northwest China to meet the national target (both 5.00%) of the Chinese Children’s Development Program 2021–2030 [34].

Our results from both descriptive and multivariate analysis demonstrated that children’s better diet quality was associated with a lower likelihood of being stunted and a higher likelihood of being reported as healthy by their caregivers. These findings are consistent with previous studies in both developed and developing countries. For example, a study conducted in Ireland among 9-year-old children found that poor diet quality was significantly associated with overnutrition problems like obesity [15], which means BmiAZ exceeds more than two standard deviations above the international means [27,29]. Another study among children under 5 years old in rural Bangladesh showed that poor diet quality was a strong predictor of stunting [20]. Meanwhile, a study in Cambodia showed that a better diet was associated with a reduction in stunting [31]. Studies in Chinese children showed that better diet quality was negatively associated with the prevalence of stunting, anemia (hemoglobin level < 110 g/L for children under 5 years old, <115 g/L for children aged 5–11, and <120 g/L for children aged 12–14 [35]), and obesity [36,37]. Moreover, improving diet quality was also shown to reduce autism disorder symptoms (which were characterized by lack of social interaction, deficits in language/communication, and routine repetitive behaviors) in preschoolers in Hong Kong, China [38]. Some studies from other low- and middle-income countries revealed that diet quality was an essential contributor to child health outcomes, including stunting, wasting, micronutrient deficiencies (such as lack of Vitamin A, Vitamin D, Iron, and so on), overweight, obesity, and nutrition-related NCDs [39,40]. Studies also show that better dietary quality has been linked with better health outcomes in adolescents, adults, and elderly people [41,42,43,44]. Taken together, these research findings suggest that improving diet quality could contribute to alleviating not only under-nutrition or over-nutrition problems but also NCDs. Such findings bear important implications for developing countries to achieve the SDG of reducing by one-third premature mortality from NCDs.

Our results also show that to achieve a better diet quality means children need to consume more healthy and diverse food groups. However, it is far from easy for every child to get access to a high-quality diet in rural remote Northwest China. On the one hand, a high-quality diet with healthy and diverse food groups can be costly for those households in the study area. As one of the poorest areas in China, the annual per capita disposable income of residents in rural Gansu Province was 9629 Yuan in 2019 (the national average was 16,021 Yuan), ranked the bottom five among all provinces of China [45]. On the other hand, caregivers’ lack of awareness and knowledge of preparing healthy and diverse food for children makes children vulnerable to malnutritional problems [46]. The results indicated that improving children’s diet quality towards reducing malnutrition problems among children needs effort in both economic and social aspects.

In addition to DQS, results from regressions also showed that some child characteristics are significantly associated with their health outcomes. Specifically, preschoolers had less risk of being underweight compared to pupils, which was consistent with previous studies suggesting that child age was positively associated with her/his likelihood of being underweight [39,47]. Compared to boys, girls had a higher risk of being underweight and having any NCD symptoms over the preceding 14 days., which is consistent with studies conducted in Bangladesh and Cambodia [48,49]. Other socio-demographic risk factors for health problems among the study sample included left-behind status and maternal education level. Children who had at least one parent migrated were at high risk of having any NCD symptoms over the preceding 14 days, but less risk of being stunted. It is not clear why left-behind status has different impacts on different health outcomes. A potential explanation may be that parental migration would bring more income to the family, which helps protect children against under-nutrition problems like stunted growth [50]. However, when parents migrate, children would suffer from the lack of parental care, which might lead to a high risk of having some NCD symptoms [51]. Some strengths of this present study are worth noting. Previous studies have linked DQS with health outcomes in adults, but the association between DQS and child health outcomes is much less understood. This is especially true in the context of remote rural areas in developing countries. Our study increases our understanding of this association by comprehensively assessing dietary quality and health outcomes among preschoolers and pupils in remote rural areas of Northwest China and examining the relationship between the two. Moreover, in addition to anthropometric measures, we also used the caregiver-reported health status as child health outcomes. Compared to height- and weight-based health outcomes, the health outcomes reported by the caregiver could give a long-term and reliable estimation of the child’s health, which provides another dimension to reflect the child’s health status from the caregiver’s perspective [52,53].

However, we also acknowledge four limitations of the present study. First, even though DQS has been widely used in the literature [13,19,39], it is not free of shortcomings. One of the criticisms of DQS is it cannot measure an individual’s habitual diet well. Previous studies show that there are huge seasonal variations in food consumption. People consume more vegetables and fruits in summer than in other seasons [54,55]. This study used a one-month recall period, as it has been shown in many diet-related studies such a recall period is less subject to recall error [17,56]. There might be other valid time frames for recall, such as the previous 6 months. Second, the relatively small sample size (n = 733 for underweight) may limit the power of the study. For example, we did not find any association between diet quality and being underweight, which is not consistent with previous studies suggesting a negative relationship between the two [57]. Therefore, the relationships are still worth further explorations with bigger sample sizes in the future. Third, the study was based on cross-sectional data. Therefore, the causal relationship between children’s dietary quality and their health outcomes cannot be established. Finally, although this study measures child health outcomes with both anthropometric data and reports by primary caregivers, more health outcomes, such as micronutrient deficiencies, nutrition-related noncommunicable diseases, and mental health should also be included in future studies.

Three policy implications could be drawn from these research findings. First, to better achieve SDG 3.4 and related goals in China, both malnutrition and NCDs among children in rural remote areas should be considered. Second, efforts are needed to improve the diet quality among preschoolers and pupils in rural remote areas of China. Compared with other interventions such as nutrition supplements or conditional cash transfers [58], increasing dietary diversity and healthy food intake through nutrition education or counseling for children and their caregivers tends to be more feasible, cost-effective, and scalable in undeveloped areas such as the site of the study. Finally, when designing and implementing child dietary interventions, special attention should be paid to girls, pupils, left-behind children, as well as children with less educated mothers.

## 5. Conclusions

The present study observed a high prevalence of stunting and being underweight among children in rural areas of Northwest China, and these children also had a high likelihood of being reported unhealthy and having NCD symptoms over the preceding 14 days by their primary caregivers. Moreover, we also found a statistically significant association between diet quality and health outcomes among children. With cross-sectional data, this study is not able to identify the causal relationship between diet quality and health outcomes among children, nor the underlying mechanisms. In the future, whenever possible, studies based on long panel data are encouraged to better understand the relationship between diet quality and health outcomes among children and the underlying mechanisms. Ideally, randomized controlled trials of diet interventions could be conducted to examine whether there exists any causal relationship between the two and the underlying mechanisms. These could be topics for future prospective studies.

## Figures and Tables

**Table 1:** Characteristics of sample children and their households (N = 1085).

Characteristics	Category	n/Mean	%/SD
DQS		28.57	11.29
	Q1	257	23.69
	Q2	177	16.31
	Q3	224	20.65
	Q4	226	20.83
	Q5	201	18.53
Gender	Boy	538	49.59
	Girl	547	50.41
Ethnic group	Han	448	41.29
	Non-Han	637	58.71
Premature status	Born normal	955	88.26
	Born premature	127	11.74
Education level	Preschool	350	32.26
	Primary school	735	67.74
Left behind status	Both parents at home	379	34.93
	At least one parent migrated	706	65.07
Time spent on TV/mobile	<60 min per day	825	76.11
	≥60 min per day	259	23.89
Mother’s education level	Illiteracy	539	49.68
	Literate but less than junior high	294	27.10
	At least junior high	252	23.22
Mother’s BMI status	Normal weight	701	64.61
	Underweight	152	14.01
	Overweight	232	21.38
Household SES	Bottom 1/3	280	25.81
	Middle 1/3	643	59.26
	Top 1/3	162	14.93
HPDS	Low HPDS (0–3)	960	88.48
	Middle HPDS (4–6)	125	11.52
	High HPDS (7–9)	0	0.00
Stunting	No	861	87.68
	Yes	121	12.32
Underweight	No	656	89.50
	Yes	77	10.50
Being reported healthy	No	295	27.19
	Yes	790	72.81
Having any NCD symptoms	No	429	39.54
	Yes	656	60.46

SD, standard error; DQS, diet quality score; Q, quintile; BMI, body mass index; SES, socioeconomic status; HPDS, household production diversity score; NCD, non-communicable disease.

**Table 2:** Child health outcomes by DQS, n (%).

DQS	Stunting	Underweight	Being Reported Healthy	Having Any NCD Symptoms
Q1	43 (19.03)	22 (12.87)	170 (66.15)	154 (59.92)
Q2	18 (11.32)	10 (9.17)	123 (69.49)	93 (52.54)
Q3	21 (10.50)	17 (11.56)	160 (71.43)	149 (66.52)
Q4	25 (12.14)	16 (10.46)	174 (76.99)	136 (60.18)
Q5	14 (7.33)	12 (7.84)	163 (81.09)	124 (61.69)
*p*-value	0.006	0.638	0.003	0.093

DQS, diet quality score; Q, quintile.

**Table 3:** Association between DQS and health outcomes, OR (95% CI).

Variables	Stunting		Underweight		Being Reported Healthy		Having Any NCD Symptoms	
	Unadjusted	Adjusted	Unadjusted	Adjusted	Unadjusted	Adjusted	Unadjusted	Adjusted
DQS	0.96 **	0.97 *	0.98	0.99	1.03 **	1.02 **	1.00	0.99 *
	**(0.95–0.98)**	**(0.95–1.00)**	(0.95–1.02)	(0.95–1.03)	**(1.02–1.05)**	**(1.01–1.03)**	(0.99–1.01)	**(0.98–1.00)**
Gender								
Boy		Ref.		Ref.		Ref.		Ref.
Girl		1.15		1.43 *		0.94		1.27 *
		(0.79–1.67)		**(1.02–2.01)**		(0.78–1.15)		**(1.03–1.58)**
Ethnic group								
Han		Ref.		Ref.		Ref.		Ref.
Non-Han		0.92		1.18		0.83		0.60
		(0.45–1.92)		(0.58–2.42)		(0.53–1.31)		(0.36–1.00)
Premature status								
Born normal		Ref.		Ref.		Ref.		Ref.
Born premature		1.60		1.44		1.06		1.08
		(0.75–3.41)		(0.70–2.97)		(0.62–1.82)		(0.75–1.56)
Education level								
Primary school		Ref.		Ref.		Ref.		Ref.
Preschool		0.77		0.59 *		1.04		1.23
		(0.38–1.55)		**(0.38–0.90)**		(0.76–1.42)		(0.92–1.65)
Left-behind status								
Both parents at home		Ref.		Ref.		Ref.		Ref.
At least one parent migrated		0.58 **		0.60		0.94		1.47 *
		**(0.39–0.85)**		(0.26–1.37)		(0.79–1.13)		**(1.04–2.09)**
Time spent on TV/mobile								
<60 min per day		Ref.		Ref.		Ref.		Ref.
≥60 min per day		0.79		0.91		1.01		1.06
		(0.56–1.13)		(0.56–1.50)		(0.80–1.27)		(0.73–1.56)
Mother’s education level								
Illiteracy		Ref.		Ref.		Ref.		Ref.
Literate but less than junior high		0.69		0.68		1.03		1.05
		(0.41–1.16)		(0.43–1.09)		(0.76–1.39)		(0.82–1.36)
At least junior high		0.57		0.63		2.31 **		1.03
		(0.28–1.18)		(0.27–1.49)		**(1.32–4.06)**		(0.70–1.52)
Mother’s BMI status								
Normal weight		Ref.		Ref.		Ref.		Ref.
Underweight		0.97		1.42		1.06		0.85
		(0.55–1.71)		(0.86–2.35)		(0.76–1.47)		(0.59–1.23)
Overweight		0.96		0.81		0.91		1.08
		(0.58–1.60)		(0.50–1.33)		(0.63–1.33)		(0.80–1.46)
Household SES								
Bottom 1/3		Ref.		Ref.		Ref.		Ref.
Middle 1/3		0.78		1.08		1.14		1.13
		(0.53–1.15)		(0.60–1.94)		(0.85–1.52)		(0.91–1.39)
Top 1/3		0.55		1.28		1.17		1.42
		(0.27–1.13)		(0.42–3.94)		(0.70–1.94)		(0.86–2.35)
HPDS								
Low HPDS		Ref.		Ref.		Ref.		Ref.
Middle HPDS		0.97		1.27		1.22		1.38
		(0.37–2.55)		(0.59–2.77)		(0.77–1.94)		(0.77–2.49)

DQS, diet quality score; OR, odds ratio; CI, confidence interval; NCD, non-communicable disease; BMI, body mass index; SES, socioeconomic status; HPDS, household production diversity score. Bold values represent that they are statistically significant; ** and * mean statistically significant at 1% and 5% level. Standard errors are clustered at the village level.

## Data Availability

The data presented in this study are available upon request from the corresponding author.
